# Analysis on the prevalence and temporal trends of adverse birth outcomes among neonates in Shanghai from 2010 to 2023

**DOI:** 10.3389/fpubh.2025.1563613

**Published:** 2025-07-18

**Authors:** Yongfa Qin, Jia Zhao, Yiyuan Li, Jing Chen, Yin Dai, Hui Li, Tao Zhang, Zhe Sun, Ying Lu, Xue Han

**Affiliations:** ^1^Department of Chronic Non-communicable Disease Control, YangPu District Center for Disease Control and Prevention, Shanghai, China; ^2^School of Public Health, Shanghai Jiao Tong University School of Medicine, Shanghai, China

**Keywords:** adverse birth outcomes, temporal trends, advanced maternal age, prevalence, correlation study

## Abstract

**Background:**

Previous research has focused on the risk factors of adverse birth outcomes and its short-term and long-term consequences. However, study on the temporal trends of adverse birth outcomes is few. Furthermore, the population-level correlation between the rate of advanced maternal age (AMA) and the prevalence of adverse birth outcomes remains underexplored. This study aimed to provide the most recent temporal trends of adverse birth outcomes in Shanghai, China, and analyze the correlation between the prevalence of AMA and the prevalence of these adverse birth outcomes.

**Methods:**

A total of 173,690 birth data was collected from four regionally influential hospitals in Shanghai from 2010 to 2023. The prevalence of adverse birth outcomes (including preterm birth, low birth weight, small for gestational age, and birth defect) was calculated. Joinpoint regression analysis was conducted to estimate the temporal trends and calculate the Average Annual Percentage Change (AAPC) and Annual Percentage Change (APC) of adverse birth outcomes and AMA. A correlation study design was employed to evaluate the population-level correlation between the prevalence of adverse birth outcomes and AMA.

**Results:**

There were 13,445 (7.74%) preterm birth (PTB), 10,226(5.89%) low birth weight (LBW), 7,152 (4.12%) small for gestational age (SGA), and 3,227 (1.86%) birth defects (BD) over the past 14 years. Sex differences were observed across different adverse birth outcomes. The prevalence of PTB (AAPC = 0.87%, *P* = 0.045) and LBW (AAPC = 2.94%, *P* < 0.001) showed significant upward trends from 2010 to 2023. The prevalence of SGA (APC = 2.42%, *P* < 0.001) presented an increasing trend from 2012 to 2023, while the prevalence of BD (AAPC = 5.73%, *P* = 0.227) remained relatively stable. The rate of AMA (AAPC = 10.14%, *P* < 0.001) also showed a significant upward trend from 2010 to 2023. Additionally, this study found a strong positive correlation between the rate of AMA and the prevalence of LBW (r = 0.89, *P* < 0.001) and BD (r = 0.92, *P* < 0.001). Moderate positive correlations were observed between AMA and the prevalence of PTB (r = 0.61, *P* = 0.022) and SGA (r = 0.75, *P* = 0.002).

**Conclusion:**

The overall prevalence of PTB, LBW, and SGA has shown an increasing trend, aside from BD. AMA also has risen annually and was significantly associated with these adverse birth outcomes. This suggests that enhancing support for advanced-age mothers could potentially mitigate adverse birth outcomes. Besides, gender differences on these adverse birth outcomes demonstrate the implementation of gender-specific healthcare strategies.

## 1 Introduction

Preterm birth (PTB), low birth weight (LBW), small for gestational age (SGA), and birth defects (BD) are the most prevalent adverse birth outcomes. These outcomes not only lead to increased rates of infant disability and mortality, but also increase the long-term risk of developing chronic diseases such as diabetes, cardiovascular diseases, chronic respiratory diseases, and neurobehavioral problems (1). They also impose a substantial economic and health burden (2). Around 13.4 million newborns are delivered prematurely, accounting for ~1 in 10 of all live births. Furthermore, nearly one million of these preterm infants die each year (3). In nearly all countries with reliable data, the prevalence of PTB is increasing while China has the second-highest number of preterm infants worldwide (4). Annually, over 20 million infants are born with LBW, constituting 15.5% of all newborns worldwide. Of these, 95.6% occur in developing countries (5). In 2010, among 135 million infants born in low- and middle-income countries, an estimated 29.7 million (22%) were SGA full-term births, and 2.8 million (2.1%) were SGA preterm births (6). According to the World Health Organization, ~12.6% of global neonatal deaths each year are associated with BDs. Around 240,000 newborns die within the first 28 days of life due to BDs, with an additional 170,000 deaths among children aged 1 month to 5 years annually (7). In China, the prevalence of BDs ranges from 0.715% to 19.184%, with ~900,000 new cases reported annually (8).

The precipitous decline in fertility rates has emerged as a pressing concern for China's national agenda (9). Moreover, in 2022, the birth rate dipped to 6.77‰, coupled with a natural population growth rate of −0.6‰, marking the first instance of negative growth since 1962 (10). The issue of declining birth rates is particularly pronounced in China's major urban clusters. As a pivotal city within the Yangtze River Delta, Shanghai now faces the dual challenges of severe aging and low birth rates, both of which threaten the sustainable development of its economy and society. Against this backdrop, efforts to prevent adverse birth outcomes are of paramount importance.

In recent years, many studies have reported various risk factors of adverse birth outcomes (1, 11–15). However, research focusing on the prevalence and temporal trends of adverse birth outcomes remains limited and incomplete (16). Analyzing the epidemiological spectrum and temporal trends of major adverse birth outcomes can provide data support for formulating targeted public health policies. Hence, this study aimed to present the most precent temporal trends of adverse birth outcomes in Shanghai, China from 2010 to 2023.Furthermore, this study explored the population-level correlation between the prevalence of AMA and adverse birth outcomes.

## 2 Materials and methods

This observational study was based on regional birth registration database. It utilized birth data from four regionally influential hospitals (Xin Hua Hospital Affiliated to Shanghai Jiao Tong University School of Medicine, XH; Margaret Williamson Red House Hospital Affiliated to Fudan University, MWRH; Changhai Hospital of Shanghai, CH; Yangpu District Central Hospital Affiliated to Tongji University, YDC) in Shanghai. The time frame of this study is from January 1, 2010 to December 31, 2024.

Joinpoint regression analysis was utilized to estimate the temporal trends in the prevalence of adverse birth outcomes and advanced maternal age over the years. In this study, we did not set a maximum number of joinpoints (i.e., locations where trends change), instead, we allowed the software [Joinpoint Regression Program (version 4.9.0)] to automatically select the optimal number of joinpoints. The Average Annual Percentage Change (AAPC) and Annual Percentage Change (APC) will automatically output by the software (17). Moreover, correlation study was used to analyze the population-level correlation between the prevalence of AMA and adverse birth outcomes.

### 2.1 Data source and quality control

Birth monitoring in Shanghai commenced in 2003, and since 2012, a comprehensive city-wide network information reporting system has been implemented. Over the past two decades, a robust workflow has been established, yielding the collection of high-quality birth registration data. Specifically, medical and healthcare institutions qualified to provide midwifery services (hereinafter referred to as “maternity hospitals”) across the city collect detailed information on every newborn delivered at their facilities. This information is recorded in birth medical record forms and promptly submitted to the “Shanghai Birth Medical Information System” for online reporting. All birth case details undergo multiple levels of review and investigation by the delivering hospital, the district CDC, and the Shanghai CDC. The information recorded in the system encompasses basic demographic data of parents, the hospital of delivery, time of birth, gestational weeks, birth weight, length, and the presence of BD. For cases involving BD, district CDC conducts monthly quality control reviews and incorporates additional cases identified through investigations by medical institutions and maternal and child health centers. Regular spot checks of hospitalized medical records are conducted, and missing birth reports are investigated twice annually in medical institutions to guarantee the completeness and accuracy of birth information.

### 2.2 Definition of adverse birth outcomes

In this study, adverse birth outcomes including PTB, LBW, BD and SGA.PTB was defined as a delivery occurring between 28 weeks and <37 weeks of gestation (18). And this definition is aligned with the *Clinical guidelines for the prevention and treatment of preterm birth (version 2024)* (19). The PTB rate was calculated as the proportion of premature births among all singleton live births during a specific period. LBW was defined as a live-born infant weighing <2,500 g at birth (20). SGA was defined as an infant whose birth weight falls below the 10th percentile by sex and gestational week of all singleton live births in a given region (21). BD, also known as congenital disorders, are a condition existing at or before birth. After birth, newborns undergo a comprehensive physical examination at the delivery hospital. Detected BD are registered and coded in accordance with the International Statistical Classification of Diseases and Related Health Problems (Tenth Revision), (ICD-10), and reported to the regional CDC monthly.

### 2.3 Statistical analysis

This study employed descriptive statistics to present the characteristics of adverse birth outcomes from 2010 to 2023.

Statistical analyses were conducted with R software (version 4.2.2) and the Joinpoint Regression Program (version 4.9.0), with a significance level set at *P* < 0.05 (two-tailed).

## 3 Results

### 3.1 Adverse birth outcomes among different hospitals from 2010 to 2023

From 2010 to 2023, a total of 173,690 newborns were reported across four hospitals, including 90,322 (52.00%) males and 83,368 (48.00%) females. The overall numbers of PTB, LBW, SGA, and BD were 13,445, 10,226, 7,152, and 3,227 cases, respectively. The distribution of these adverse birth outcomes reported by each hospital is summarized in Table 1.

**Table 1:** Adverse birth outcomes in different institutions from 2010 to 2023.

**Hospital**	**Adverse birth outcomes**			
	**PTB (n,%)**	**LBW (n,%)**	**SGA (n,%)**	**BD (n,%)**
XH	4,204 (10.38)	3,366 (8.31)	1,861(4.59)	2,005 (4.95)
MWRH	7,235 (6.85)	5,552 (5.26)	4,225 (4.00)	1,052 (0.99)
YDC	514 (4.55)	360 (3.19)	508 (4.50)	90 (0.80)
CH	1,492 (9.15)	948 (5.81)	558 (3.42)	80 (0.49)
**Total**	13,445 (7.74)	10,226 (5.89)	7,152 (4.12)	3,227 (1.86)

### 3.2 Analysis on differences of adverse birth outcomes among newborns from 2010 to 2023

**Figure 1:**
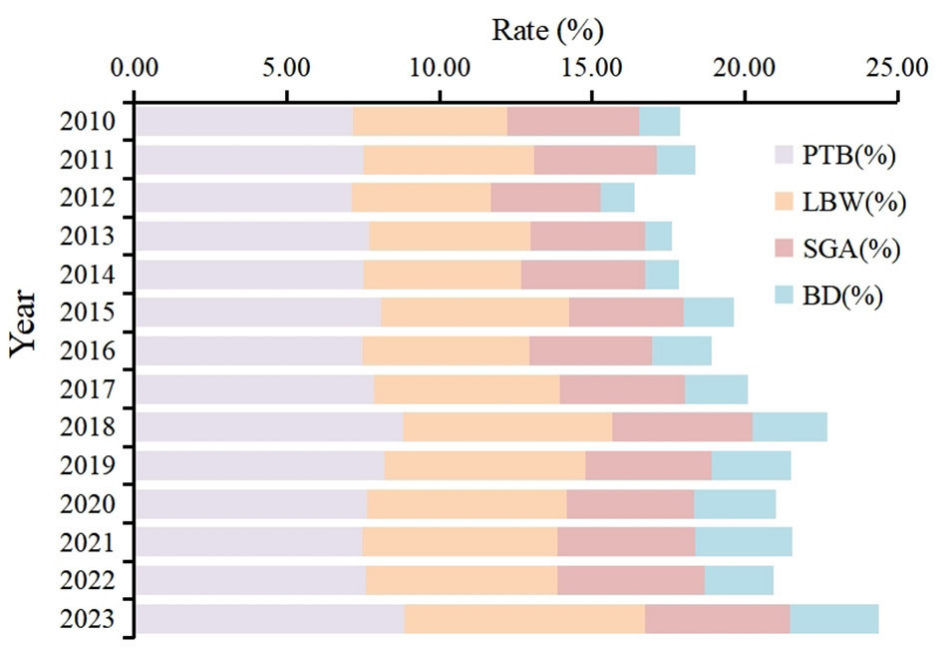
The changes in the pattern of adverse birth outcomes from 2010 to 2023.

The analysis revealed significant differences in adverse birth outcome rates across different years (χ^2^ = 361.25, *P* < 0.001; Figure 1). From 2010 to 2023, there were significant gender differences in the rates of PTB (χ^2^ = 110.52, *P* < 0.001), LBW (χ^2^ = 15.803, *P* < 0.001), BD (χ^2^ = 78.404, *P* < 0.001), and SGA (χ^2^ = 544.84, *P* < 0.001; Table 2). The overall proportion of male newborns with PTB (8.12 vs. 7.33%) and BD (2.06 vs. 1.63%) was higher compared to females. Conversely, the overall proportion of female newborns with LBW (6.37 vs. 5.44%) and SGA (5.47 vs. 2.87%) was higher than that observed in males.

**Table 2:** Gender distribution of adverse birth outcomes from 2010 to 2023.

**Year**	**PTB**		**LBW**		**SGA**		**BD**	
	**Female (n, %)**	**Male (n, %)**	**Female (n, %)**	**Male (n, %)**	**Female (n, %)**	**Male (n, %)**	**Female (n, %)**	**Male (n, %)**
2010	380 (57.58)	280 (42.42)	228 (48.82)	239 (51.18)	150 (37.78)	247 (62.22)	68 (54.40)	57 (45.60)
2011	470 (54.59)	391 (45.41)	319 (49.45)	326 (50.55)	176 (38.26)	284 (61.74)	89 (61.38)	56 (38.62)
2012	576 (52.41)	523 (47.59)	321 (45.92)	378 (54.08)	188 (33.81)	368 (66.19)	97 (57.74)	71 (42.26)
2013	595 (53.08)	526 (46.92)	353 (45.72)	419 (54.28)	195 (35.65)	352 (65.35)	76 (59.84)	51 (40.16)
2014	662 (52.96)	588 (47.04)	387 (44.59)	481 (55.41)	230 (34.07)	445 (65.93)	124 (66.31)	63 (33.69)
2015	588 (54.34)	494 (45.66)	424 (51.15)	405 (48.85)	186 (36.91)	318 (63.09)	120 (54.30)	101 (45.70)
2016	699 (54.61)	581 (45.39)	453 (47.94)	492 (52.06)	255 (37.23)	430 (62.77)	181 (53.87)	155 (46.13)
2017	640 (55.60)	511 (44.40)	443 (49.61)	450 (50.39)	224 (37.46)	374 (62.54)	173 (57.10)	130 (42.90)
2018	611 (55.14)	497 (44.86)	418 (48.49)	444 (51.51)	205 (35.41)	374 (64.59)	180 (59.21)	124 (40.79)
2019	562 (54.62)	467 (45.38)	393 (47.24)	439 (52.76)	202 (38.92)	317 (61.08)	198 (60.37)	130 (39.63)
2020	442 (56.59)	339 (43.41)	316 (46.88)	358 (53.12)	148 (34.50)	281 (65.50)	163 (59.71)	110 (40.29)
2021	364 (52.75)	326 (47.25)	295 (49.75)	298 (50.25)	147 (35.17)	271 (64.83)	164 (55.97)	129 (44.03)
2022	380 (56.97)	287 (43.30)	275 (49.73)	278 (50.27)	145 (34.12)	280 (65.88)	111 (56.06)	87 (43.94)
2023	363 (54.50)	303 (45.50)	287 (48.32)	307 (51.68)	138 (38.33)	222 (61.67)	121 (55.25)	98 (44.75)
**χ** ^2^	110.52		15.803		544.84		78.404	
***P***	<0.001		<0.001		<0.001		<0.001	

### 3.3 Temporal trends of adverse birth outcomes from 2010 to 2023

In this study, the temporal trend of the prevalence of PTB and LBW showed a significant upward trajectory from 2010 to 2023 (AAPC = 0.87%, *P* = 0.045; AAPC = 2.94%, *P* < 0.001; Table 3). Further, the prevalence of PTB increased from 7.16% in 2010 to 8.83% in 2023, while the prevalence of LBW rose from 5.07% to 7.88%. For SGA, the overall temporal trend from 2010 to 2023 was relatively stable (AAPC = 0.74%, *P* = 0.496). While from 2012 to 2023, the prevalence of SGA showed an upward trend (APC = 2.42%, *P* < 0.001). The prevalence of BD showed a relatively stable trend from 2010 to 2023 (AAPC = 5.73%, *P* = 0.227).

**Table 3:** Temporal trends of adverse birth outcomes from 2010 to 2023.

**Year**	**PTB (%)**	**LBW (%)**	**SGA (%)**	**BD (%)**
2010	7.16	5.07	4.31	1.36
2011	7.49	5.61	4.00	1.26
2012	7.13	4.54	3.61	1.09
2013	7.69	5.29	3.75	0.87
2014	7.49	5.20	4.04	1.12
2015	8.06	6.18	3.75	1.65
2016	7.45	5.50	3.99	1.96
2017	7.85	6.09	4.08	2.07
2018	8.81	6.85	4.60	2.42
2019	8.18	6.61	4.12	2.61
2020	7.61	6.56	4.18	2.66
2021	7.45	6.41	4.52	3.16
2022	7.57	6.28	4.83	2.25
2023	8.83	7.88	4.77	2.90
***AAPC***	0.87	2.94	0.74	5.73
***t***	2.24	5.38	0.68	1.21
***P***	0.045	<0.001	0.496	0.227

### 3.4 Temporal trend of AMA rate and its correlation with adverse birth outcomes

The rate of AMA showed a significant upward trend from 2010 to 2023 (AAPC = 10.14%, *P* < 0.001). During the period from 2010 to 2014, the rate of AMA remained relatively stable (APC = 7.07, *P* = 0.005). Subsequently, the AMA rate increased sharply at an average annual rate of 26.91% (*P* = 0.036) between 2014 and 2017, followed by a moderate increase of 4.57% (*P* = 0.025) from 2017 to 2023.

**Figure 2:**
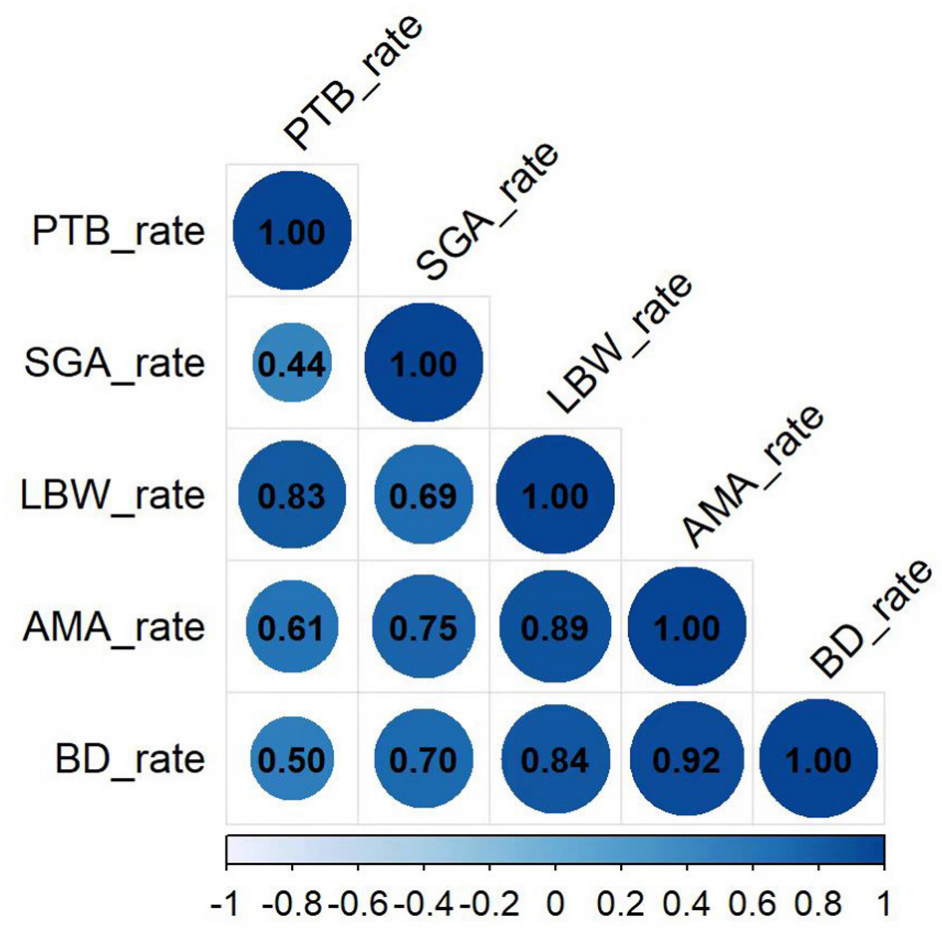
The correlation coefficient matrix between the rate of AMA and adverse birth outcomes.

Further analysis revealed a strong positive correlation between AMA rate and the rates of LBW (*r* = 0.89, *P* < 0.001) and BD (*r* = 0.92, *P* < 0.001; Figure 2). Moderate positive correlations were also observed with the rates of PTB (*r* = 0.61, *P* = 0.022) and SGA (r = 0.75, *P* = 0.002).

## 4 Discussions

This study analyzed birth data of 173,690 neonates from four hospitals in Shanghai from 2010 to 2023.The findings revealed significant average annual increases in the prevalence of PTB (0.87%), LBW (2.94%) and SGA (2.42%, from 2012 to 2023) over the study period. While the rate of BD remained relatively stable. Moreover, a population-level correlation was observed between AMA and the prevalence of these adverse birth outcome.

In this study, the total PTB rate was 7.74% (ranging from 7.16 to 8.83%), which is comparable to the rates observed in middled-income countries (9.3%) and lower than the global prevalence of 9.3–12.6% (22). Generally, high-income countries report lower rates of PTB. For example, a study from England reported that the prevalence of PTB in England was 6.1% between 2015 and 2017 (23). Globally, the rate of PTB is on the rise. In this study, the rate of PTB increased at an average annual rate of 0.87%, consistent with global trends. For example, Chile showed that the PTB rate increased from 5.0% in 1992 to 7.2% in 2018, with an average annual rate of 1.44% (24). In this study, PTB was defined as a gestational age between 28 and 37 weeks, whereas many studies define it as <37 weeks (25). Varying definitions of PTB across studies may complicate direct comparisons. However, this difference was unlikely to significantly affect the overall temporal trend. Its noteworthy that from 2016 to 2022, PTB-related complications accounted for up to 16.6% (12.4–16.6%) of all-cause deaths in children under 5 years old (26). Therefore, the rising prevalence of PTB will present a significant public health challenge that should not be overlooked. Moreover, factors such as changes in the lifestyle of women of childbearing age, environmental exposures, the spectrum of pregnancy-related diseases, and pregnancy intervals following adjustments to fertility policies have also been evolving during the study period. These changes may influence the long-term trends of PTB (27). Therefore, future study could use an age-period-cohort model to better assess temporal trends in PTB (28).

LBW, the second leading cause of morbidity and mortality in newborns, and childhood morbidity, following PTB (29). In this study, the overall rate of LBW was 5.89% (ranging from 4.54 to 7.88%), which is relatively close to the rates reported in the United Kingdom (6.8%) and South Korea (6.6%), but lower than Japan (9.4% in 2019) and the United States (8.3%) (30). It is alarming that the prevalence of LBW in China is increasing at an average rate of 2.94%. In Japan, a trend analysis from 2000 to 2019 showed an initial increase in LBW prevalence during the first decade, followed by a decline in the second decade (31). Such variations across countries may be attributed to differences in maternal and child health care quality, prenatal care, obstetric interventions, and dietary habits (32).

LBW is often associated with PTB, as preterm delivery frequently results in low birth weight. Both conditions share modifiable risk factors, including AMA. Previous study confirmed that AMA was an independent risk factor for PTB (33). While another study reported a similar increasing trend in LBW and prevalence of AMA (34). In this study, the population-level correlation of AMA and PTB (*r* = 0.61), LBW (*r* = 0.89) was observed. Although, the population-level correlation found in the present study can't be equally to the casual relationship, the result also underscored the potential adverse effect of AMA on birth outcomes.

Compared to the definitions of preterm infants (considering only gestational age) or low birth weight infants (considering only weight), the definition of SGA (Small for Gestational Age) integrates both gestational age and birth weight factors. Due to its comprehensive information, SGA has garnered increasing attention in recent years for its implications in neonatal health (35). Globally, SGA accounts for over 17% of all live births, with prevalence ranging from 3 to 10% (36). Approximately two-thirds of SGA cases occur in Asia (37). In this study, the overall rate of SGA was 4.12% (range from 3.61% to 4.83%), and an upward trend was observed over the years. Research on the temporal trends of SGA is still relatively scarce, but the growing body of evidence highlights its significant impact on neonatal and long-term health outcomes. Existing research indicates that compared to newborns with appropriate for gestational age (AGA) birth weights (including both term and preterm infants), SGA infants are at significantly higher risks for perinatal complications and mortality. Furthermore, it has been reported that SGA is implicated in neurocognitive developmental impairment, adverse perinatal outcomes (such as thermoregulation and immune function disorders), neonatal asphyxia, hypoglycemia, hypocalcemia, and polycythemia (38). This suggests that when evaluating child health related outcomes, greater attention should be paid to SGA.

The association between SGA and AMA has been inconsistent. Some studies have indicated a direct association between SGA and AMA (39–41), while other studies suggest that the effect of AMA on SGA risk may vary depending on the studied population (42). In this study, we found a significant population-level correlation between SGA (*r* = 0.75) and AMA. However, some potential confounding factors may influence the robustness of this correlation. Nevertheless, the correlation observed in this study still provided new evidence regarding the impact of AMA on SGA. Besides, BDs are complex and diverse. Considering that analyzing each type of BD separately may result in small sample sizes, which could affect the robustness of temporal trend, this study did not categorize BDs into specific types. Analysis of specific types of defects could be conducted in future multicenter studies with larger sample sizes. Globally, the prevalence of BDs is estimated at 2–3%, while in China, it ranges from 4 to 6% (43, 44). In this study, the overall rate of BDs in Shanghai was found to be 4.12% (ranging from 3.61 to 4.83%), consistent with national estimates. Although the temporal trend of BDs remained relatively stable over the past 14 years, they have consistently posed a significant healthcare issue due to their long-term resource requirements and their status as a major cause of perinatal mortality and infant mortality. The stable trend in the Shanghai population may be attributed to abundant medical resource. high-quality medical service, socioeconomic status, and education background of pregnant women. Moreover, the above factors have been identified as significantly associated with BDs (8). This study found a strong population-level correlation between BDs (*r* = 0.92) and AMA. A large-scale analysis of 1.26 million delivery records in China also revealed an increased risk of BDs among older mothers (45). Similarly, a study conducted in Brazil found that, even after adjusting for maternal education levels and local development conditions, AMA remained associated with a higher risk of BDs (46). Compared to the aforementioned individual-level studies, our correlation study cannot establish a causal association between AMA and BDs. However, there is a growing consensus that AMA increase the risk of BDs.

Consistent with previous studies, this research observed significant gender differences in adverse birth outcomes. Prevalence of PTB and BD was significantly higher in male infants compared to female infants, while the rates of LBW and SGA were higher in female infants. A study conducted in Japan indicated that male fetal gender was associated with an increased risk of preterm delivery, very preterm delivery, and extremely preterm delivery (47). Furthermore, female fetuses exhibit significantly slower growth rates than male fetuses; for example, the mid-pregnancy head circumference of female fetuses is smaller than that of male fetuses. In terms of intrauterine growth restriction, it is also more prevalent among female fetuses (48). These findings were corroborated by a multicenter, large-sample study conducted across 39 hospitals in China (49). These results highlight the importance of developing gender-specific healthcare strategies to address adverse birth outcomes.

It is well-known that AMA significantly increases the risk of adverse pregnancy outcomes and delivery complications (50, 51). The observed trend of increasing AMA in this study can be partly attributed to the Chinese government's 2015 announcement that replaced the longstanding one-child policy with a universal two-child policy (52). Sociocultural shifts, including delayed marriage, singlehood, and child-free lifestyles among younger generations, further exacerbate this trend (53, 54). In this study, compared to gender factor, AMA seemed to be a better approach for reducing the risk of adverse birth outcomes. In general, increased risk of adverse birth outcomes caused by AMA can be mitigated through societal efforts. The following measures could be beneficial: (1) Strengthen public education campaigns to raise awareness about the optimal timing for childbirth and the risks associated with age, encouraging women to conceive at suitable ages. (2) Provide enhanced preconception counseling programs for women of advanced age to address reproductive risks related to aging and offer genetic screening options before pregnancy. (3) Develop prenatal care plans for older pregnant women, including more frequent monitoring and targeted nutritional guidance. (4) Improve access for women of advanced age to assisted reproductive technologies and offer appropriate counseling regarding success rates and potential risks.

This study utilized birth monitoring data to analyze the temporal trends of adverse birth outcomes in Shanghai, China. Offering unique insights into the population-level correlation between AMA and adverse birth outcomes. However, the study also has several limitations: (1) Due to this observational study extracts from a birth registration database, certain clinical information could not be obtained. For example, parity, gravidity, maternal nutritional status, and pregnancy complications could not be controlled. And these factors could have potential influence on the temporal trend, especially on the correlation between AMA and adverse birth outcomes. (2) China is a vast country, and as a mega-city, Shanghai possesses medical resources and social support that may exceed those found in other provinces. Results from Shanghai population may underestimate the strength of the association between AMA and adverse birth outcomes, as the risks associated with AMA may be more pronounced in areas with limited medical resources. The high-quality healthcare services available in Shanghai may better manage the risks of AMA pregnancies, which may not be accessible in areas with fewer resources. These facts could affect the generalizability of the findings to the broader Chinese context. Especially, for those regions with limited medical resources and a low proportion of AMA. Moreover, during the study period Shanghai has experienced significant socio-economic changes. Higher education and income levels are associated with delayed childbirth as well as better access to healthcare, which may confound our results. Future studies should incorporate more diverse medical institutions and regions, including rural hospitals and primary healthcare facilities, to obtain more representative results. (3) The correlation observed between AMA and adverse birth outcomes based on population level, potential confounders are inevitable. These unmeasured factors significantly limit our ability to interpret the observed correlation as direct effects of AMA. Future research should use individual-level data with comprehensive adjustment for these factors to clarify the complex relationship between AMA and adverse birth outcomes. Nevertheless, we believe that analyzing trends in adverse outcomes and the relationship between delayed childbearing and adverse outcomes is still highly meaningful, as it can provide a basis for the formulation of public health policies.

## 5 Conclusion

This study systematically analyzed the temporal trends of adverse birth outcomes in Shanghai from 2010 to 2023, highlighting a continuous increase in PTB, LBW, and SGA rates. The findings emphasize a population-level correlation between AMA and these outcomes, as well as significant gender differences in adverse birth outcomes. These findings align with global and domestic researches, underscoring the necessity of targeted public health interventions. While the data from Shanghai cannot be directly generalized to represent the entire nation of China, it still provides valuable insights for maternal and child health care in other major metropolitan areas with similar characteristics to Shanghai.

As fertility rates decline, reducing adverse birth outcomes, particularly among the growing population of advanced-age mothers, is a crucial public health priority. Future strategies should focus on improving prenatal care access and quality for older mothers, alongside broader societal support measures. Additionally, larger-scale studies are recommended to investigate the underlying reasons for rising adverse birth outcomes rates and to evaluate the effectiveness of intervention strategies, providing a robust foundation and data support for the formulation and implementation of public health policies.

## Data Availability

The original contributions presented in the study are included in the article/supplementary material, further inquiries can be directed to the corresponding author.
